# Self-rated amygdala activity: an auto-biological index of affective distress

**DOI:** 10.1017/pen.2019.1

**Published:** 2019-06-13

**Authors:** Katherine E. MacDuffie, Annchen R. Knodt, Spenser R. Radtke, Timothy J. Strauman, Ahmad R. Hariri

**Affiliations:** 1 Department of Speech and Hearing Sciences, University of Washington, Seattle, WA, USA; 2 Department of Psychology and Neuroscience, Duke University, Durham, NC, USA; 3 Department of Psychiatry and Behavioral Sciences, Duke University, Durham, NC, USA

**Keywords:** auto-biology, functional MRI, amygdala, subjective well-being, psychopathology

## Abstract

Auto-biological beliefs—beliefs about one’s own biology—are an understudied component of personal identity. Research participants who are led to believe they are biologically vulnerable to affective disorders report more symptoms and less ability to control their mood; however, little is known about the impact of self-originating beliefs about risk for psychopathology, and whether such beliefs correspond to empirically derived estimates of actual vulnerability. Participants in a neuroimaging study (*n* = 1256) completed self-report measures of affective symptoms, perceived stress, and neuroticism, and an emotional face processing task in the scanner designed to elicit threat responses from the amygdala. A subsample (*n* = 63) additionally rated their own perceived neural response to threat (i.e., amygdala activity) compared to peers. Self-ratings of neural threat response were uncorrelated with actual threat-related amygdala activity measured via BOLD fMRI. However, self-ratings predicted subjective distress across a variety of self-report measures. In contrast, in the full sample, threat-related amygdala activity was uncorrelated with self-report measures of affective distress. These findings suggest that beliefs about one’s own biological threat response—while unrelated to measured neural activation—may be informative indicators of psychological functioning.

Understanding the biological basis of human behavior has long been of interest to the scientific community and the general public. Indeed, biological explanations for behavior appeal to the human tendency to “essentialize” or categorize people based on what are perceived to be fundamental, underlying natural characteristics (Haslam, 2011; Haslam & Ernst, 2002; Rothbart & Taylor, 1992). This tendency is particularly powerful in the arena of mental health. Biological explanations for disorders like depression or schizophrenia—while initially promoted to reduce blame and stigma against affected individuals—have had the paradoxical effect of increasing stigma, in part by implying the biological “otherness” of those affected (Haslam & Kvaale, 2015). Essentialist attitudes are present within the mental health community as well: clinicians in one study reported less empathy towards potential patients after being given a biological explanation for their symptoms (Lebowitz & Ahn, 2014).

Whether such biological essentialism extends to attitudes about one’s own biology—or *auto-biology*—is less clear (Lebowitz, 2014). The term auto-biology, as we use it here, refers to one’s knowledge, attitudes, or beliefs about one’s own biological systems (MacDuffie & Strauman, 2017). Prior studies have employed false feedback to individuals about their own biology to test the impact of auto-biological beliefs on mental health. For example, participants with a history of depression were shown results from a fictional “Rapid Depression Test,” conducted via cheek-swab (Kemp, Lickel, & Deacon, 2014). Those who received results describing a serotonin imbalance were more pessimistic about their mood-regulation abilities and prognosis compared to participants who received “normal” results. Similarly, participants who were told via a fictional test that they were genetically susceptible to depression reported higher retrospective ratings of depressive symptoms compared to participants who were told they were not genetically susceptible (Lebowitz & Ahn, 2017). Providing fictional feedback about biological vulnerability, therefore, appears to increase essentialist thinking about one’s own biology and risk for mental illness.

To our knowledge, no studies have examined auto-biological beliefs about vulnerability to mental illness directly. We aimed to capture auto-biological beliefs relevant to mental health and explore their association with self-reported distress and with an individual’s actual biology. We investigated beliefs about a specific biological signature—threat-related amygdala activity (TRA)—which is associated with risk for stress-related psychopathology but is measurable in the general population (Swartz, Knodt, Radtke, & Hariri, 2015). After learning about TRA as a risk biomarker, participants were asked to rate their own amygdala activity. Participants’ self-ratings of amygdala activity (hereafter SRA) were subsequently compared to their actual TRA as measured by BOLD fMRI. Finally, we explored the associations between SRA, TRA, and commonly used self-report measures of personality and affective distress. Data were analyzed in an exploratory manner, with the goal of determining whether beliefs about one’s own biological vulnerability for affective disorders correspond to actual neural activity, and the relationship of each to self-reported distress.

## 1. Methods

### 1.1 Participants

#### 1.1.1 Full sample

Participants were young-adult university students enrolled in the Duke Neurogenetics Study (DNS)—a large-scale investigation capturing a wide range of behavioral and biological traits, with the goal of understanding how neurogenetic and experiential factors interact to predict psychopathology in early adulthood. Participants were recruited via on-campus advertising, and informed consent was obtained in accordance with the Duke University School of Medicine Institutional Review Board. Exclusion criteria for the DNS included: (1) medical diagnoses of cancer, stroke, head injury with loss of consciousness, untreated migraines, diabetes requiring insulin treatment, chronic kidney or liver disease, or lifetime history of psychotic disorder; (2) use of psychotropic, glucocorticoid, or hypolipidemic medication; and (3) conditions affecting cerebral blood flow and metabolism (e.g., hypertension). A total of 1256 participants (713 female, mean age = 19.7 years) were included in the current analyses. All had complete self-report data on included measures and fMRI data that met quality control standards (see below).

#### 1.1.2 Self-rating subsample

Sixty-three participants (42 women, mean age = 19.8 years) also took part in the amygdala self-rating sub-study. The racial/demographic characteristics of the subsample were representative of the full DNS sample.

### 1.2 Threat-related amygdala activity (TRA)

Our widely utilized face-matching task has been used extensively to elicit robust amygdala activity across an array of experimental protocols and sample populations (Barch et al., 2013; Bertolino et al., 2005; Meyer-Lindenberg et al., 2009; Miller et al., 2016; Tesli et al., 2013).

**Figure 1.**
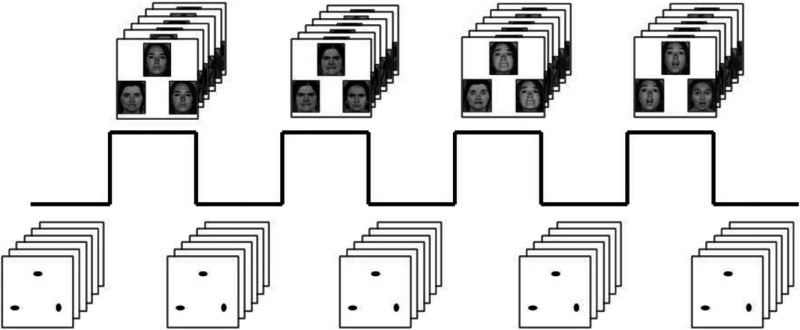
Emotional face processing task. Experimental blocks of a perceptual face-matching task alternate with control blocks of a shape-matching task. In each, participants are instructed to select which of the two stimuli on the bottom of the screen matches the top stimulus.

The task consists of four experimental blocks interleaved with five control blocks. In the DNS version of this task, there is one experimental block each of fearful, angry, surprised, and neutral facial expressions presented in a pseudorandom order across participants. During these experimental blocks, participants view a trio of faces and select one of two faces (on the bottom) identical to a target face (on the top, see Figure 1). Each of these blocks consists of six images, balanced for gender, all of which were derived from a standard set of pictures of facial affect (Ekman & Friesen, 1976). During the control blocks, participants view a trio of simple geometric shapes (circles and vertical and horizontal ellipses) and select one of two shapes (bottom) that are identical to a target shape (top). Each of these blocks consists of six different shape trios. All the blocks are preceded by a brief instruction (“Match Faces” or “Match Shapes”) that lasts 2 s. In the experimental task blocks, each of the six face trios is presented for 4 s with a variable interstimulus interval (ISI) of 2–6 s (mean = 4 s) for a total block length of 48 s. A variable ISI is used to minimize expectancy effects and resulting habituation and maximize amygdala reactivity throughout the paradigm. In the control blocks, each of the six shape trios is presented for 4 s with a fixed ISI of 2 s for a total block length of 36 s. Total task time is 390 s.

#### 1.2.1 BOLD fMRI data acquisition

Each participant was scanned using one of two identical research-dedicated GE MR750 3 T scanners equipped with high-power high-duty-cycle 50-mT/m gradients at 200 T/m/s slew rate, and an eight-channel head coil for parallel imaging at high bandwidth up to 1 MHz at the Duke-UNC Brain Imaging and Analysis Center. A semi-automated high-order shimming program was used to ensure global field homogeneity. A series of 34 interleaved axial functional slices aligned with the anterior commissure-posterior commissure plane were acquired for full-brain coverage using an inverse-spiral pulse sequence to reduce susceptibility artifacts (TR/TE/flip angle = 2000 ms/30 ms/60; FOV = 240 mm; 3.75 × 3.75 × 4 mm voxels; interslice skip = 0). Four initial radiofrequency excitations were performed (and discarded) to achieve steady-state equilibrium. To allow for spatial registration of each participant’s data to a standard coordinate system, high-resolution three-dimensional structural images were acquired in 34 axial slices coplanar with the functional scans (TR/TE/flip angle = 7.7 s/3.0 ms/12; voxel size = 0.9 × 0.9 × 4 mm; FOV = 240 mm, interslice skip = 0).

#### 1.2.2 BOLD fMRI data preprocessing

Anatomical images for each subject were skull-stripped, intensity-normalized, and nonlinearly warped to a study-specific average template in the stereotactic space of the Montreal Neurological Institute using the ANTs open-source registration tools (Klein et al., 2009). BOLD time series for each subject were processed in AFNI (Cox, 1996). Images for each subject were despiked, slice-time-corrected, realigned to the first volume in the time series to correct for head motion, coregistered to the anatomical image using FSL’s Boundary Based Registration (Greve & Fischl, 2009), spatially normalized into MNI space using the nonlinear warp from the anatomical image, resampled to 2 mm isotropic voxels, and smoothed to minimize noise and residual difference in gyral anatomy with a Gaussian filter, set at 6-mm full-width at half-maximum. All transformations were concatenated so that a single interpolation was performed. Voxel-wise signal intensities were scaled to yield a time series mean of 100 for each voxel. Volumes exceeding 0.5 mm frame-wise displacement or 2.5 standardized DVARS (Nichols, 2017; Power et al., 2014) were censored from further analyses.

#### 1.2.3 fMRI quality assurance criteria

Quality control criteria for inclusion of a participant’s fMRI data were: >5 volumes for our condition of interest (see below) retained after censoring for FD and DVARS and sufficient temporal SNR within the bilateral amygdala, defined as >3 standard deviations below the mean of this value across participants. The amygdala was defined anatomically using a high-resolution template generated from 168 Human Connectome Project datasets (Tyszka & Pauli, 2016). Additionally, data were only included in further analyses if the participant demonstrated sufficient engagement with the task, defined as achieving at least 75% accuracy during the face matching condition.

#### 1.2.4 BOLD fMRI data analysis

Following preprocessing, the AFNI program 3dREMLfit (Cox, 1996) was used to fit a general linear model for first-level fMRI data analyses. A linear contrast employing canonical hemodynamic response functions was used to estimate effects of threatening faces (Angry + Fearful block > Control blocks) for each individual. These individual contrast images were then used in second-level random effects models in SPM12 (http://www.fil.ion.ucl.ac.uk/spm) accounting for scan-to-scan and participant-to-participant variability to determine mean condition-specific regional responses using one-sample t-tests. A statistical threshold of *p* < 0.05, FWE corrected across our amygdala region of interest (Tyszka & Pauli, 2016), and ≥10 contiguous voxels were applied to the contrast of interest. This was performed for the entire amygdala (all 10 subnuclei in the Tyszka atlas), the combined Basolateral nuclei, the combined Central and Medial nuclei, and the Central nucleus alone (as this subregion is only 5 voxels in each hemisphere, the 10 contiguous voxel criteria was relaxed), and single subject parameter estimates were extracted from the resulting significant functional clusters.

### 1.3 Self-rated amygdala activity (SRA)

**Figure 2.**
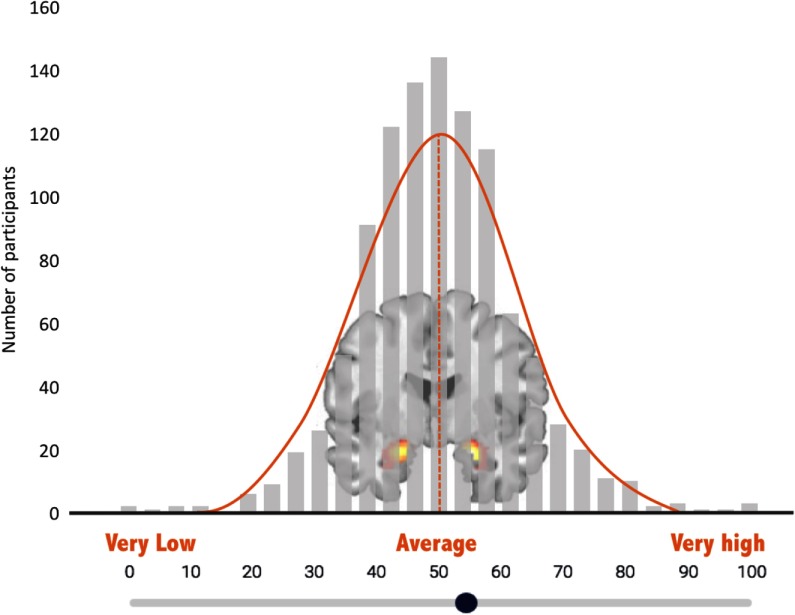
Amygdala self-rating scale. Participants used a slide to indicate their own perceived level of amygdala reactivity on this “amygdala bell curve”—a histogram depicting bilateral threat-related amygdala activity from other study participants.

The amygdala self-rating was collected after participants completed the fMRI task. Participants watched a short video, developed for the current study, which described TRA as a marker of vulnerability for emotional disorders (narration text of the video is provided as Appendix A). Comprehension of the video was assessed via two quiz questions, which were answered accurately by 100% of participants. Participants were then shown a histogram of TRA values from >1000 previous DNS participants and asked to rate themselves in reference to this “amygdala bell curve” (Figure 2), using a sliding scale from 0 to 100, with 50 representing average amygdala activity.

### 1.4 Self-report questionnaires

Data from five self-report measures of affective symptoms and/or distress were included in the current analyses: the Center for Epidemiologic Studies Depression Scale-Revised (CESD; Eaton, Smith, Ybarra, Muntaner, & Tien, 2004), a 20-item screening instrument for depression; the Mood and Anxiety Symptom Questionnaire (MASQ; Watson et al., 1995), a 77-item measure assessing depressive, anxious, and mixed symptomatology; the Perceived Stress Scale (PSS; Cohen, Kamarck, & Mermelstein, 1983), a 10-item measure of the degree to which participants perceive their lives as stressful (i.e., unpredictable and uncontrollable); the State-Trait Anxiety Inventory-Trait version (STAI-T; Spielberger, 1983), a 20-item instrument widely used to measure trait anxiety; and the NEO-Revised Personality Inventory (Lord, 2007), a 243-item measure that yields scores across five domains of personality—the Neuroticism scale (NEON) was selected for the current analysis, given its relevance to psychopathology (Tackett & Lahey, 2017). Self-report measures were completed either on the same day as the fMRI scan or within 1 week.

### 1.5 Data analytic strategy

Analyses were designed to sequentially address three questions: (1) How does TRA relate to affective distress in the broader DNS sample? (2) How does SRA relate to TRA? and (3) Does SRA relate to affective distress after accounting for TRA? By necessity, all analyses using the SRA measure were tested on the subsample who provided this rating. Outlier analyses revealed one SRA outlier with a rating >3 standard deviations below the sample mean; with this individual excluded, the final subsample size for SRA analyses was 62. Analyses of associations between TRA and self-report measures of affective distress were conducted on the full DNS sample (*n* = 1256).

All results were FDR corrected for multiple comparisons. Associations between TRA for the left and right hemispheres and each self-report measure were tested with individual regression models. Associations between SRA and self-report measures were tested with regression models controlling for mean TRA. Finally, all the analyses that included SRA were tested with both linear and quadratic regression models; due to the design of the amygdala self-rating, we sought to test the possibility that associations with SRA could be linear and/or parabola-shaped (i.e., as perhaps belief in “difference from average,” whether above or below, could be associated with subjective distress and/or TRA). Linear vs. quadratic models were compared by adding an orthogonal quadratic term to the regression equations and assessing for improvement in model fit. All analyses were conducted in R (R Core Team, 2017). Analysis code is publicly available at http://faculty.washington.edu/kmacd/SRAmy_analysis.html.

## 2. Results

### 2.1 TRA and affective distress

Associations between left and right hemisphere TRA and each of five self-report measures (NEON, PSS, STAI-T, CESD, and MASQ) were tested across the entire sample (*n* = 1256) and corrected for multiple comparisons. None of the self-report measures were significantly associated with left or right TRA (all *p*s > 0.5). This suggests that, across the entire sample, measured threat-related amygdala reactivity did not correspond to self-reported affective symptoms, perceived stress, or neuroticism.

### 2.2 SRA and TRA

Associations between TRA and SRA were tested in the subsample that completed the amygdala self-rating protocol (*n* = 62). SRA was not associated with left or right TRA (Left: adjusted *R* ^2^ = −0.02, *F*[1,60] = 0.0005, *p* = 0.98; Right: adjusted *R* ^2^ = −0.02, *F*[1,60] = 0.01, *p* = 0.91) suggesting that participants’ rating of their own amygdala activity did not correspond to their actual TRA measured in the scanner. Adding the quadratic term did not improve fit for either model (Left: *F*[1,59] = 0.23, *p* = 0.63; Right: *F*[1,59] = 0.22, *p* = 0.64). Given that there were no laterality effects in the first two analysis steps, and the high correlation between left and right TRA across the whole sample (Pearson’s *r* = 0.76, *p* < 0.0001), an averaged bilateral amygdala reactivity value was calculated across hemispheres and used for the final analysis step.

### 2.3 SRA and affective distress (accounting for TRA)

**Figure 3.**
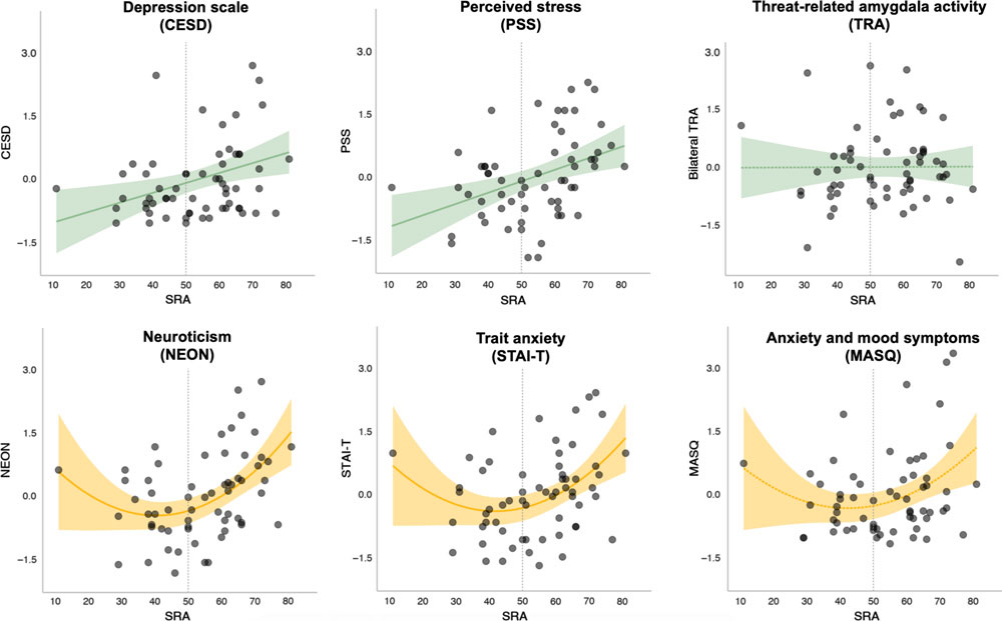
Amygdala self-rating and self-reported affective symptoms/distress. Depicted in green are models that showed a linear relationship between SRA and the outcome variable, which did not improve with addition of a quadratic term. Depicted in yellow are models that improved with addition of quadratic term. Dotted lines represent models that did not meet the significance threshold (*p* > .05). Shaded area represents a 95% confidence interval.

Associations between SRA and each of the five self-report measures were tested, controlling for mean TRA. In four of the five models, SRA significantly predicted the outcome (NEON, PSS, STAI-T, and CESD) over and above TRA and after correcting for multiple comparisons; one model showed an association at trend-level (MASQ). When the quadratic term was added to the model, the fit for NEON (*F*[1,58] = 7.1, *p* = 0.009) and STAI-T (*F*[1,58] = 6.3, *p* = 0.01) showed significant improvement. In contrast, the model fit for PSS (*F*[1,58] = 2.5, *p* = 0.12) and CESD (*F*[1,58] = 2.4, *p* = 0.12) was not significantly improved with the addition of the quadratic term. Parameter estimates for the full models (including both linear and quadratic SRA terms, as well as TRA) are presented in Table 1, and plots of SRA and each outcome measure are shown in Figure 3. These results suggest that while a linear model was the best fit for predicting perceived stress (PSS) and depressive symptoms (CESD) from SRA and TRA, a quadratic fit was better for predicting trait anxiety (STAI-T) and neuroticism (NEON). The model predicting affective (i.e., both anxious and depressive) symptoms on the MASQ showed a trend towards fit improvement with inclusion of the quadratic term (*F*[1,58] = 4.0, *p* = 0.05). However, even with the quadratic term the full model for MASQ failed to reach significance (see Table 1).

**Table 1.** Regression results

	Linear SRA *β*	Quadratic SRA *β*	Bilateral TRA *β*	Full Model adj. *R* ^2^	*F* Statistic (df)
NEON	2.74 (0.91)**	2.42 (0.91)**	−0.02 (0.12)	0.18	5.45 (3,58)**
PSS	3.04 (0.92)**	1.46 (0.92)	−0.08 (0.12)	0.15	4.67 (3,58)**
STAI-T	2.21 (0.93)*	2.34 (0.93)*	0.10 (0.12)	0.13	4.11 (3,58)*
CESD	2.63 (0.95)**	1.48 (0.95)	−0.02 (0.12)	0.12	3.41 (3,58)*
MASQ	1.76 (0.97)^†^	1.93 (0.97)^†^	−0.02 (0.12)	0.07	2.47 (3,58)^†^

Parameter estimates and model statistics for models predicting self-report scores from self-rated and actual threat-related amygdala activity (SRA and TRA). Models included orthogonal linear and quadratic SRA predictors.

Numbers in parentheses are standard errors, unless otherwise indicated.

* *p* < 0.05, ** *p* < 0.01, ^†^ *p* < 0.1 (trending).

## 3. Discussion

To our knowledge, this study represents the first time that auto-biological beliefs have been compared to an individual’s own brain function—in this case, threat-related amygdala activity during an emotional face matching task. We found no significant correspondence between self-rated and measured amygdala activity within the constraints of available statistical power. The observed association between self-rated amygdala reactivity and perceived stress and depressive symptoms was linear, suggesting that participants who believed their amygdala activity was higher than average were likely to report more stress and symptoms of depression. However, trait anxiety and neuroticism showed a quadratic association with SRA, suggesting that participants who rated themselves as further from average activity, in either direction, tended to be more anxious and prone to negative affect. The finding that belief in auto-biological “difference-from-average” was a robust predictor of negative affectivity is consistent with prior evidence that feeling different from one’s peer group is associated with self-reported psychopathology (Allan & Gilbert, 1995).

These data and prior work suggest that auto-biological beliefs may be a useful indicator of psychological functioning. Indeed, self-rated amygdala activity, while unrelated to actual TRA, predicted affective distress across a variety of self-report measures. The fact that a self-rating predicted other self-report measures is perhaps unsurprising; however, the variety of affective domains predicted by SRA (perceived stress, depressive symptoms, trait anxiety, and neuroticism) is noteworthy given that it was a single item querying beliefs about one's own biology. If replicated in further studies, the auto-biological measure developed here may prove to be an efficient and revealing index of subjective distress.

Emerging evidence from the physical (rather than mental) health literature suggests that auto-biological beliefs can impact actual physiology. Turnwald and colleagues (2019) recently found that telling individuals that they were at high vs. low genetic risk for obesity (via either cardiorespiratory exercise capacity or physiological satiety) impacted not only their subjective ratings of exercise and fullness, but also their actual exercise and satiation-related physiology (i.e., metabolic gas exchange, ventilatory flow rate, and glucagon-like peptide-1 response). In some cases, an individual’s beliefs appeared to influence their physiological response more strongly than actual genetic risk status (Turnwald et al., 2019). While similar physiological impacts of auto-biological beliefs about mental illness have yet to be demonstrated, these results are consistent with prior work showing the negative emotional impact of learning in an experiment that one is biochemically or genetically at risk for depression (Kemp et al., 2014; Lebowitz & Ahn, 2017). Taken together, these data suggest that auto-biological beliefs may involve similar mechanisms as the placebo and nocebo effects, with appreciable (and potentially clinically significant) consequences for subjective and objective health outcomes (Finniss, Kaptchuk, Miller, & Benedetti, 2010; Kaptchuk & Miller, 2015).

Our results could be interpreted as reflecting a disconnect between participants’ beliefs about their auto-biology and their actual, measurable biological response to threatening stimuli. However, the results could also reflect a methodological weakness in the reliability of ROI-based analyses of task-based functional neuroimaging data. An increasing number of studies report that amygdala activity measured with tasks that use emotional facial expressions as stimuli—including the task used in our protocol—yields poor test–retest reliability (Lipp, Murphy, Wise, & Caseras, 2014; Lois, Kirsch, Sandner, Plichta, & Wessa, 2018; Nord, Gray, Charpentier, Robinson, & Roiser, 2017; Plichta et al., 2012; Sauder, Hajcak, Angstadt, & Phan, 2013), and many prior published associations between TRA and genetic markers have failed to replicate (Avinun, Nevo, Knodt, Elliott, & Hariri, 2017). These findings highlight a critical challenge for individual-differences neuroimaging research: the difficulty of predicting trait-like differences in psychopathology from neural responses that show limited reliability over time and are susceptible to state influences (Dubois & Adolphs, 2016; Sauder et al., 2013). Indeed, our observation that SRA significantly predicted a range of self-report measures of emotional distress but actual TRA did not, even in the much larger full study sample, adds further support to the notion that single-timepoint, task-elicited activation values from ROIs may not be optimal predictors of trait-like individual differences.

Additional limitations of this exploratory research are worth noting. The sample size for the amygdala self-rating was small, and ratings were made via a single item. Firm conclusions about the utility of self-rated auto-biological measurements as indices of emotional distress or other aspects of personality will depend on further testing with larger samples and an expanded set of items. While the video narration was intended to be emotionally neutral (see Appendix A), it is possible that the video itself created a negative mood state, which could have impacted participants’ ratings. The finding that some of the self-report measures showed a linear and others a quadratic relationship with SRA was unexpected; extrapolating beyond the observed patterns to conclude a difference between the constructs based on the linear vs. quadratic relationship would be speculative at this point. Finally, our auto-biological rating contained an embedded social comparison, as participants were asked to rate themselves in reference to DNS peers. For our participants—undergraduates in a competitive academic environment—such comparison may have been particularly salient (Allan & Gilbert, 1995). Future iterations should attempt to disentangle beliefs about auto-biology from comparison to peers.

## 4. Conclusion

How individuals think about their own biology is an important yet understudied topic, particularly when considered in relation to mental health. The results from this study suggest that, in a sample of university students, perceiving oneself as biologically different from peers is associated with negative affectivity and emotional distress. The specificity of these findings to auto-biological ratings (vs. social comparison in general) remains to be determined. However, these preliminary findings contribute to the growing evidence that reductive biological explanations for mental disorders can be harmful, increasing stigma and prognostic pessimism in patients, clinicians, and the general public alike (Haslam & Kvaale, 2015; Lebowitz, 2014; Lebowitz & Ahn, 2014). Auto-biological beliefs are poised to gain increasing relevance as mental health research and treatment become more biologically informed (MacDuffie & Strauman, 2017). Understanding an individual’s beliefs about his or her own biological vulnerabilities is an important first step towards correcting maladaptive beliefs that could hinder treatment seeking and recovery.

## Appendix A. Narration of video describing TRA and introducing the amygdala self-rating

Hello! Welcome to our experiment on face processing and emotion. Thank you for participating. You have been asked to participate in this experiment because you are enrolled in the Duke Neurogenetics Study, or DNS for short. As part of the DNS study, you completed a task in the MRI scanner in which you had to match pictures of faces, some of which were expressing negative emotions such as anger and fear. While you were completing that task, we were collecting data from an area of your brain called the amygdala.

The amygdala is a small, almond-shaped structure near the center of your brain that is responsible for processing emotions, particularly those associated with threat (such as anger or fear). We collected information on how your amygdala responded to facial expressions depicting anger and fear, and then we combined that information with a number of other genetic, personality, and cognitive measures that we collected.

The reason that we are interested in how your amygdala responds to angry and fearful faces is because studies from our lab and others have shown that amygdala reactivity is a marker of vulnerability for emotional disorders such as depression and anxiety. Furthermore, we know that people with a particular genetic profile have higher amygdala reactivity.

This graph [Figure 2] shows amygdala reactivity to fearful and angry faces in the sample of more than 1000 undergraduates who have participated in the DNS study thus far. The higher the bar, the more participants are represented. You can see that this graph is in the shape of a bell curve, with most students falling in the average range and a few with very low or very high amygdala reactivity. Judging from your own sense of yourself, your moods, and your responsiveness to emotions, where do you think you fall on this graph? We’ll pause briefly now so you can advance to the next page. There you can mark clearly, using the slider, where you think your own amygdala reactivity falls on this distribution from very low to very high.
